# Self-Determination as a Psychological and Positive Youth Development Construct

**DOI:** 10.1100/2012/759358

**Published:** 2012-04-30

**Authors:** Eadaoin K. P. Hui, Sandra K. M. Tsang

**Affiliations:** ^1^Division of Learning Development and Diversity, Faculty of Education, The University of Hong Kong, Hong Kong; ^2^Department of Social Work and Social Administration, Faculty of Social Sciences, The University of Hong Kong, Hong Kong

## Abstract

This paper presents a review of self-determination as a positive youth development construct. The definition and conceptualization of the concept are examined from the perspective of self-determination theory and the functional theory of self-determination. Theories of self-determination from the perspective of motivation and skills enhancement are examined. Factors contributing to self-determination, such as autonomy-supportive teaching and parenting style, culture, efficacy of intervention programmes, and the educational benefits of self-determination for students, are discussed. Strategies to promote self-determination in an educational context and implications for further research and practice are discussed.

## 1. Introduction

Adolescence is a critical phase of life during which young people face physical, psychological, intellectual, and emotional concerns and challenges, search for self-identity, explore new roles, and deal with transition to secondary schools and later from school to work and adulthood. Individuation and separation are processes that adolescents have to go through. Achieving independence and autonomy, setting personal goals and making plans, and acquiring values and ethics are developmental tasks that all adolescents have to realize. Being self-determined is a developmental task that all young people have to confront and is pertinent to their whole-person development. 

## 2. Definition of Self-Determination

Self-determination, as a psychological construct, refers to volitional actions taken by people based on their own will, and self-determined behaviour comes from intentional, conscious choice, and decision [1]. The conceptualization and definition of self-determination varies according to its theoretical orientations. The self-determination theory (SDT) proposed by Deci and Ryan [2], for example, focuses on the motivational aspect of self-determination and the role of self-determined motivation and autonomy on students' learning and education [3]. Self-determination is defined as “the capacity to choose and to have those choices….be the determinations of one's action” (page 38) [4].

In the field of special education with youth and adults with disabilities, researchers focus more on the development of cognitive, social and behavioural components which are essential dispositional characteristics for self-determined behaviour. Wehmeyer [5], for example, refers self-determined behaviour as “volitional actions that enable one to act as the primary causal agent in one's life and to maintain or improve one's quality of life” (page 117). Self-determination is defined as skills, knowledge, and beliefs, which facilitate goal-directed, self-regulated, and autonomous behaviour [6].

In the context of positive youth development, self-determination is defined as “the ability to think for oneself and to take action consistent with that thought” (page 105) [7]. Self-determination of young people is fostered through positive youth development programmes, which target at promoting autonomy, independent thinking, self-advocacy, empowerment of young people, and their ability to live according to values and standards. Such conceptualization is in line with the emergence of positive psychology which emphasizes fostering of human strengths [8].

In short, people who are self-determined are self-initiated, self-directed, and make things happen in their lives. Self-determination is about the competence of young people in engaging in volitional behaviour and their autonomy in making choices and decisions, which are nurtured in supportive social environments.

### 2.1. Self-Determination from the Human Motivation Perspective

#### 2.1.1. Theoretical Framework of SDT

SDT is based upon the assumption that human persons are active and growth-oriented agents, inclined to organize and initiate their actions with reference to their values and interests, with the tendency to integrate social norms and practices, intrinsically motivated to pursue personal goals, and striving to master the environments. The development of these tendencies and qualities is dependent upon the kind of support they receive from the socializing environments, which may promote or undermine their intrinsic motivation and internalization [2, 9].

SDT postulates that the satisfaction of the three basic psychological needs, namely, competence, relatedness, and autonomy, is pertinent for the optimal development and functioning of human persons. Competence refers to having the feeling of being capable to meet the demands of environments and face daily challenges. Such need can be fulfilled by the experiences of enacting and achieving desired goals and having effective outcomes. Autonomy is about being volitional and self-endorsing in one's behaviour and having the control to make choices from one's own will. The need for autonomy differs from being independent, selfish, and having freedom of choices [2, 10]. The essential elements which facilitate autonomy include self-awareness of one's motives, emotions, and external demands, having active involvement, and having the chances for self-direction and choice making. Satisfaction of the need for autonomy at home and in a school environment is likely to facilitate the development of intrinsic motivation and internalization [2]. In addition, both the needs for competence and autonomy are necessary and essential for the maintaining of intrinsic motivation [11]. Relatedness is about the need to achieve a sense of closeness, connectedness, and belongingness with others. The satisfaction of the need for relatedness will provide emotional security for further exploration. Feelings of closeness to the significant others such as parents and teachers will facilitate the process of internalization of values, social norms, and practice. Hence, socioemotional relatedness is pertinent to internalization and the subsequent motivation and self-regulation to engage in tasks demanded by others [2, 11].

#### 2.1.2. Self-Determination and Educational Outcomes

Research studies have provided evidence that support for students' psychological needs for autonomy, competence, and relatedness facilitates autonomous self-regulated learning, academic performance, and well-being [11]. High levels of autonomy, relatedness, and competence are associated with more satisfying learning experiences [3]. Academic achievement is strongly associated with autonomous motivation [12]. Young people who are regulated by autonomous and intrinsic motivation experience more positive educational outcomes at schools. For example, students who were autonomously motivated had higher academic achievement, self-esteem, perceived competence, personal control, and creativity [13] and showed a more adaptive learning attitude, and academic success [14]. Students taught by autonomy-supportive teachers were found to have increased intrinsic motivation, higher competence and self-esteem, more interest for lessons, greater creativity, flexibility in thinking and conceptual understanding, and more active involvement in information processing than were their counterparts whose teachers were controlling [13, 15, 16]. Autonomous motivation was also found to be associated with psychological well-being [17]. Research studies have shown that autonomously motivated students reported more positive affect and emotions, having more enjoyment of academic work, experiencing greater life and school satisfaction, and having lower ill-being such as depression [11, 12]. In addition, higher autonomy in schools is associated with lower dropout rates, lower level of anxiety, more positive coping strategies [18]. Students whose environments are supportive of their needs have a greater tendency to engage in learning, which promotes hope [19].

#### 2.1.3. Factors Contributing to the Development of Self-Determination

**Parenting Styles**

According to SDT, the social contexts that are responsive and supportive can facilitate young people to engage in self-initiated, self-regulated, and volitional behaviour [2]. Parents in the context of family play a very important role in the cultivation of self-determination. First, parents who meet their children's needs for autonomy contribute to their self-regulation and motivation. Research studies have provided evidence that parents who are autonomously supportive provide their children with choices and options and allow them to explore and enact according to their own interests and values [20, 21]. By showing genuine interest to their children's needs and being empathic to their views and perspectives [22], parents help their children to develop themselves as active and volitional agents. Research by Soenens and Vansteenkiste [9] has shown that parental autonomy support contributed significantly to self-determination in the domain of school and peer relationship. On the other hand, a controlling parental style which focuses on outcome rather than process and on controlling techniques tend to undermine children's intrinsic motivation and internalization [23, 24]. Second, the provision of structure by parents, such as giving clear expectation about behaviour, promotes children's competence, understanding of ways to attain success, and perceived personal control [24]. Third, parental involvement facilitates children's motivation to achieve, internalization of values, and students' academic self-regulation [24, 25]. A caring and supportive home environment also satisfies children's needs for relatedness. In short, parental autonomy support, structure, and involvement are pertinent to fostering autonomous self-regulation in children.

**Teacher Autonomy-Supportive Style**

SDT suggests that teacher autonomy support and structure are pertinent to help students to attain optimal learning. Autonomy support and structure, though different, are student focused and positively related. Teachers who provide students with structure and guidelines tend to have a more autonomy-supportive style [26]. Research studies have found positive relationships between teacher autonomy support, and students' scholastic self-determination, school engagement and school adjustment [9]. Autonomy-supportive teachers, similar to autonomy-supportive parents, contribute to students' self-determination through offering choices, providing rationale for choices, empathizing with students' perspectives, and minimizing the use of controlling language in the classroom environments [26]. Autonomy-supportive teachers also identify, cultivate, and develop students' inner motivational resources [27]. These practices provide students with the opportunity to pursue personal goals and interest and to satisfy their needs for autonomy and competence.

In addition, an autonomy-supportive learning environment contributes to the enhancement of students' perceived competence, interest, and enjoyment [28]. Students with a low autonomy level benefit particularly in an autonomy-supportive environment, where they learn to be more autonomous and self-regulated, leading to improvement in learning performance.

**Culture and Self-Determination**

SDT posits that the needs for competence, autonomy, and relatedness are innate, universal, and compatible. Hence, fulfilment of these needs contributes to the optimal functioning of all individuals across cultures and societies [29]. SDT acknowledges that people are influenced by their culture in assigning meaning and interpretation to their autonomous experience as positive or negative, to be supported or to be prevented [3]. Individuals' expressions of their needs for competence, autonomy, and relatedness may differ within cultures that hold different values. Yet, they reckon that the benefits of self-determination and the negative consequences of being nonautonomous are across culture. Cross-cultural psychologists, however, argue that the constructs of self-determination and autonomy are influenced by western cultural values. For example, autonomy is considered as a value upheld in individualist societies [30], reflecting an independent view of self [31]. Hence, the need for autonomy is in conflict with the need for relatedness and interdependent relationships cherished in collectivistic societies [32, 33]. However, other researchers argue that autonomy from the SDT perspective is about being volitional in one's act, which is different from asserting independence from significant others and having freedom of choices. In a collectivist culture, the need for autonomy can be met through internalization of the demands of others and self-endorsement of the choices [10]. A recent research by Hui et al. [34] has demonstrated that these three psychological needs are pertinent to academic motivation in the East as well as in the West. Competence was found to be the most significant predictor of academic motivation among Chinese students. Following competence, relatedness with parents was salient in predicting academic motivation. Autonomy had a strong positive association with relatedness, revealing that the higher autonomous support the students perceived from their parents, the greater the connection they felt with their parents. Another study with Chinese students from the People's Republic of China also illustrated the benefits of autonomous academic motivation to adaptive learning attitudes, academic success, and well-being [14].

### 2.2. Self-Determination from the Perspective of Skills Enhancement

#### 2.2.1. Theoretical Framework and Approaches

According to the functional theory of self-determination, people act as causal agents who make things happen. Actions that are self-determined are related to the function they serve. The essential characteristics of self-determined actions include that the person acts autonomously and in a self-realizing manner, the behaviour is self-regulated, and the act is a self-initiated response to events in a psychologically empowered manner [35]. As the functional theory of self-determination adopts a person-environment interaction framework in its conceptualization, the development of self-determination is influenced by both individual dispositional characteristics as well as environmental experiences. The ecological model of self-determination, on the other hand, considers attaining personal control over one's life as the ultimate goal of self-determination [36]. According to this model, the skills, knowledge, and beliefs that a person holds interact with the environment to facilitate the attainment of goals and desirable outcomes.

Promoting self-determination has been a major concern for youth with disabilities. Research studies have suggested that youth with disabilities lack skills, knowledge, and beliefs, which are important for their self-determination [37]. Further, students with disabilities are less self-determined than their peers without disabilities. Hence, fostering self-determination has been a major issue in the field of special education and has become best practice in secondary education and transition service [38].

In recent years, the emergence of positive psychology has had considerable impact on the field of positive youth development. Grounded in developmental theories such as Erikson's identity development theory and Bowlby's attachment theory, the positive youth development approach emphasizes identifying young peoples' strengths and competencies. The approaches are grounded in humanistic psychology, which emphasizes individuals' potentials and capabilities. It can be seen that the assumptions of humanistic theories are very similar to those that self-determination is based on, for example, emphasizing individuals' subjective awareness of themselves and others, and individuals having choice and capability for self-actualization [4]. Self-determination is one of the fifteen psychological constructs to be taught as skill development for youth with or without disabilities positive youth development programmes and Project P.A.T.H.S in Hong Kong Schools [7].

#### 2.2.2. Factors Contributing to Self-Determination of Students with Disabilities

The development of self-determination, according to functional theory, is influenced by an individual's capacity, that is, the personal characteristics and the environmental factors and instructional experiences. Regarding personal characteristics, intelligence was found to have a positive relationship with self-determination, in which individuals high in IQ scores have higher self-determination scores [39].

Research examining the effect of gender on self-determination has been limited and has produced mixed findings. Gender was not found to be significant in the study by Wehmeyer and Garner [40]. Other studies, however, found gender to assert effect on self-determination (Nota et al., 2007 and Shogren et al., 2007 cited in [41]). External factors, such as choice opportunity rather than intelligence, were found to be the primary predictor of self-determination among people with intellectual disabilities [40]. The living and working environments contribute to self-determination, with people in community-based settings having greater autonomy and more choice opportunities, whereas people from restrictive settings were lower in self-determination [42]. A recent research by Lee et al. [41] found that instructional, knowledge, and dispositional factors were stronger predictors of students' self-determination than personal factors such as age, gender, and intelligence level. Self-efficacy and outcome expectancy, student-directed transition planning instruction, and students' preintervention transition planning knowledge were strong predictors of students' determination. 

Environmental factors which contribute to self-determination include provision of self-determined role models, self-determination skill instruction and support, opportunities for choice to make decision, positive communication patterns within the school institutions and personal relationships, and provision of student support by teachers and peers [43]. In addition, developing supportive relationships with others, including teachers and peers, contribute to supporting self-determination [44]. The sense of relatedness provides security for young people to be self-determined [17]. Hence, supportive relationships encouraged by peer support programs, like peer tutoring, peer counselling, help promote self-determination [44].

#### 2.2.3. Intervention Programmes to Promote Self-Determination

Research has shown that the possession of self-determination skills is associated with improved educational outcome in school and with postschool success for youth and adults with disabilities. For example, improved self-determination skills were crucial to academic performance and success and contributed to increased class participation and postsecondary involvement [45]. Self-determination skills lead to improved outcomes in independence and employment as well as in quality of life [46].

Hence, self-determination as a construct becomes an important aspect in education and has been used widely in education programmes for students with disabilities [42]. Various models and approaches have been developed to enhance their skills in self-determination [47–53]. Most intervention programmes target at teaching skills in decision making, choice making, self-advocacy, self-efficacy, self-awareness, and self-evaluation of goals and plans [54].

 Field et al. [55], for example, developed the *Steps to Self-determination Curriculum *based on the five major components: Know yourself, Value yourself, Plan, Act, Experience Outcome, and Learn. The first two components, Know yourself and Value yourself, are about fostering self-knowledge and self-awareness. The components Plan and Act are about acquiring specific skills. Experience Outcome and Learn refer to evaluating of goals and plan and celebrating of success. This curriculum is based on the view that possession of inner knowledge of what one wants and the skills to attain the desired goals are pertinent to self-determination. Individual characteristics such as self-awareness are the building blocks for self-determination, whereas ability to set goals is the outcome of self-determination. Environmental factors, such as opportunities for choice making and the attitudes of others, also contribute to self-determination. This is an example of a comprehensive curriculum that can be used with secondary students with and without disabilities. The self-determination knowledge and skills can be integrated across subject areas to be supported at all levels in school.

The Self-Determined Learning Model of Instruction put forward by Wehmeyer et al. [56] targets at strengthening the components of self-determination, in which teachers guide students through a three-phase instructional process, namely, goal setting, taking action, and adjusting the goal or plan. In each phase, students learn to respond to problems by posing and answering a series of four questions critically in a problem-solving process, setting goals to meet their needs, making modifications and application of their self-selected goals, and adjusting actions to complete their plans. Teachers provide a set of objectives for each question and educational support in each phase to facilitate students to be self-directed learners. Hence, students act as active agent in making decisions and choices and taking actions. This approach of teaching self-regulated problem solving can be applied across a wide range of content areas for students with and without disabilities. The programme had positive effects on students' self-regulation and achievement of self-selected goals.

Since acquiring self-determination skills facilitates all adolescents, with or without disabilities, to be self-directed learners having personal control of their life, curricula which target at enhancing the components of self-determination mentioned above can be infused into the general curriculum so that all students may benefit. In addition, inclusion of youth with disabilities in mainstream education is a global trend. Deliberate infusion of self-determination instruction and development into the general curriculum will allow students with disabilities to have access to the intervention in the inclusive classrooms [54].

## 3. Strategies to Promoting Self-Determination

Theoretical approaches like SDT, functional theory, and ecological models all consider the social context as an important factor in facilitating or undermining self-determination. School is a significant social context where self-determination of students can be fostered as strength. Promoting self-determination should be a primary educational goal for all students, with or without disabilities [48]. SDT posits that students are better able to internalize their motivation and engage in self-regulated learning when their psychological needs for autonomy, competence, and relatedness are supported in their school environments. It is pertinent to provide students with opportunities to learn and apply skills to become self-regulated learners.

### 3.1. Ways to Facilitate Self-Determination Skills through Education Programmes

Adolescents, with or without disabilities, will benefit from intentional interventions which promote self-determination [1, 47]. Students can be taught systematically to develop self-determination skills through schools' guidance programmes, life education programmes, or individual learning programmes for students with disabilities and special needs. The curricula that target at enhancing self-determination skills may include activities to develop skills in goal setting, planning, evaluating and monitoring, and choice making. The curriculum units on self-determination of the Project P.A.T.H.S. are an example of developing self-determination skills systematically through a formal educational curriculum [57]. In addition, learning tasks can be structured to encourage exploration of possibilities, reasonable risk taking, and problem solving. Further, activities which target at enhancing students' self-esteem and self-confidence, appreciation of their strengths and knowledge of their limitations, and promoting self-advocacy will further facilitate students' self-understanding and communication. Through learning the self-regulatory skills of decision making, problem solving and action planning, students' personal control over their learning is likely to increase. They are better able to apply self-determination skills to their personal goals, and become more autonomous learners and self-determined persons [54]. In addition, schools can promote self-determination through integrating the components of self-determination skills into the general curriculum, with emphasis on helping students to apply self-determination skills in identifying personal goals, action planning, and evaluating. Such intentional efforts to promote self-determination is very much in line with the rationale of positive youth development, that is, helping all students to build assets and strengths, leading to the benefits of reduction in at risk behaviour.

### 3.2. Ways to Promote Autonomous Supportive Environments

Research studies have shown that students' perceived autonomy support in the classrooms leads to more positive academic outcomes [11]. Students who find their learning environment supportive of their needs for competence, autonomy, and relatedness will have greater engagement in learning, which exert influence on their psychological adjustment [19]. Teachers who are autonomy-supportive also experience a greater personal achievement, psychological needs satisfaction and well-being, and less emotional strain [27]. A student-directed learning environment where students feel respected and connected with their teachers and peers will lead to a satisfaction of the need for relatedness.

Research studies have demonstrated that the following strategies are important in promoting an autonomous supportive classroom environment, which help nurture students' inner motivational resources [27, 54]. First, teachers may consider, incorporate, and prioritize students' perspectives in learning activities, welcome students' ways of feeling, thinking and acting, and accept that students are capable of autonomous self-regulation and setting personal goals. This will mean that teachers need to find ways to engage students' interests and to introduce tasks that will challenge their competence. Second, it is important to provide explanations and rationales why certain behaviour is worth engaging in so as to facilitate students' internalization and increase their effort to engage. It is also important to make reference to the benefits of self development (i.e., intrinsic goals) rather than social image, financial success (i.e., extrinsic goals) when asking students to follow their request. Third, provision of a structure and guidance such as classroom expectations, and positive feedback are pertinent in helping students see the association between their behaviour and the outcomes. Fourth, teachers should create conditions in which students learn to take risks, make choices, and evaluate their choices and actions [54]. Fifth, patience and trust are necessary in allowing students to learn at their own pace. This will require teachers to listen to students' perspectives, to offer help when students' get stuck, to encourage students' initiatives and to provide time for self-paced learning. Sixth, accepting students' negative emotions and affects and letting students feel that teachers genuinely like, respect, and value them will enhance the students' relationship with teachers, which is critical in satisfying their psychological needs for relatedness as postulated by SDT. Lastly, provision of peer support to help foster supportive relationships and having peers as well as teachers to act as role models is critical for fostering self-determination.

## 4. Further Direction for Research and Practice in Self-Determination

Chambers et al. [42] point to the following four areas which need further research and practice for promoting self-determination. First, in the area of teacher training and support, there is a need to prepare teachers to acquire knowledge and skills in attending to the psychological needs of their students' for competence, relatedness, and autonomy, the component skills of self-determination, and the instruction strategies to foster an autonomy-supportive classroom environment and facilitate students to be self-regulated learners. This will have implications for teacher education training at both preservice and in-service levels. Further research can be directed at examining the needs and competency of teachers in promoting self-determination. Second, there is a need to have systematic implementation strategies in schools. While self-determination skills and components can be taught through specialized programmes, integrating these curriculum packages with the general curriculum has the advantage of providing access to students with or without disabilities. Such curriculum infusion has the benefit of promoting self-determination as a whole-school approach to guidance for the whole-person development of all students [58]. Further research may examine the effectiveness of the infusion of self-determination in academic curriculum. Third, parental involvement in promoting self-determination is needed, since an autonomous parental attitude relates to children's adjustment at school [23]. Strategies in promoting self-regulated learning and an autonomy-supportive environment can be disseminated to parents via workshops, seminars, and school-home collaboration projects. In addition, component skills in self-determination, such as having children identify goals, action planning, and evaluating can be introduced to parents. Research can further examine the effectiveness of this form of family involvement. Lastly, as self-determination is a developmental task, the promotion of self-determination skills needs to begin early in primary schools.
